# A Global Review of Animal–Visitor Interactions in Modern Zoos and Aquariums and Their Implications for Wild Animal Welfare

**DOI:** 10.3390/ani9060332

**Published:** 2019-06-08

**Authors:** Neil D’Cruze, Sophie Khan, Gemma Carder, David Megson, Emma Coulthard, John Norrey, Georgina Groves

**Affiliations:** 1 World Animal Protection, 222 Gray’s Inn Rd., London WC1X 8HB, UK; gemmacarder@yahoo.co.uk; 2 School of Science and Environment, Manchester Metropolitan University, All Saints Building, All Saints, Manchester M15 6BH, UK; d.megson@mmu.ac.uk (D.M.); e.coulthard@mmu.ac.uk (E.C.); j.norrey@mmu.ac.uk (J.N.); 3 Wild Welfare, 63 Queenswood Road, London SE23 2QR, UK; sophie@wildwelfare.org (S.K.); georgina@wildwelfare.org (G.G.)

**Keywords:** conservation, education, tourism, wildlife, Word Association for Zoo and Aquariums

## Abstract

**Simple Summary:**

This study explores the characteristics of animal-visitor Interactions (AVIs) (interactions between people and captive wild animals) in zoos and aquaria across the globe. We reviewed information provided on public websites of institutions that are either direct members of the World Association for Zoos and Aquariums (WAZA) or belong to regional and national associations that have WAZA membership. The opportunity for visitors to interact with wild animals was promoted on the majority of the facilities’ websites. Petting captive wild animals was the most common AVI activity advertised (43%) of facilities, and interaction with mammals was the most advertised taxonomic class (53%). Some activities involving direct contact with wildlife were promoted more commonly than expected (for example, hand feed and ride wild animals, and walk with or swim through wild animal enclosures). Some of the advertised AVIs have the potential to impact animal welfare; in light of this, we provide recommendations to balance and manage captive wild animal welfare in AVIs with other primary interconnected goals.

**Abstract:**

We provide an initial insight into the occurrence and characteristics of animal-visitor interactions (AVIs) involving captive wild animals within zoos and aquaria. Using information provided online via official public websites of modern zoos and aquaria, we found that AVIs were provided by the majority of facilities. Our study revealed that a variety of AVI types were being offered. Globally, petting captive wild animals was the most prevalent AVI type advertised (*n* = 1241 observations, 43% (534) of facilities) and Mammalia was the most advertised taxonomic class (*n* = 5142; 53% (2739)). We found certain AVI types that were more commonly offered than predicted. These were opportunities to: (1) Hand feed captive wild animals in Asia, North America and Oceania; (2) ride wild animals in Europe and North America; (3) walk with or swim through wild animal enclosures in Asia; and (4) walk with wild animals in Asia and Europe. Given the global prevalence of AVIs in modern zoos and aquaria, and an apparent lack of animal welfare focused research, we provide recommendations to help effectively balance and manage captive wild animal welfare with other primary interconnected goals.

## 1. Introduction

Modern zoos and aquaria can play an important positive role in conserving wildlife, for example, by caring for individual animals in captivity as part of wild release program [1]. However, zoos can only maintain a limited number of endangered species [2]. Zoo and aquaria remain under scrutiny for their collection paradigms, breeding and reintroduction programs in relation to conservation outputs. Most modern zoos and aquaria have a remit comprised of five primary, interconnected goals: (1) Conservation; (2) education; (3) research; (4) animal welfare; and (5) entertainment [3]. While some modern zoos and aquaria can place a major emphasis on the first four goals, a substantial number of visitors come, at least in part, for entertainment [3]. Therefore, providing entertaining experiences can encourage initial visits and subsequent returns, both of which can translate into greater revenue to help achieve its other goals [3].

Unfortunately, modern zoos and aquaria often encounter conflicts among these goals [3]. For example, one of the many reasons people have been drawn to zoos is for the opportunity to interact with less familiar animals [4] and the possibility of having direct and indirect physical contact with a wild animal can increase the appeal of a zoo or aquaria for many visitors [4,5]. In certain scenarios, depending on a range of different factors (including but not limited to the type of activity, the wild animal species, the history of an individual wild animal, and its familiarity with a given visitor involved [6]), such interactions can potentially provide positive experiences for wild animals in captivity [7]. Yet, advances in knowledge about animal welfare science have also added to some pre-existing concerns about the potential negative impact such activities can have on both the psychological and physiological wellbeing of the wild animals involved [8,9,10].

In a recent review, Hosey and Melfi [7] found that, since the 1980s, the largest contributions to the broader study of human–animal interactions (HAI) have come from the companion and agricultural contexts. However, they also drew attention to the fact that some of the perspectives from those studies have more recently been adopted in studies focused on the “more emergent field” of wild animals in zoos and aquaria (e.g., References [6,7]). In recognition of the fact that a lack of precise definitions has hindered progress in this field [7], in this study, we used the term animal–visitor interaction (AVI) (in a similar manner as Reference [4]) to refer to categories of activities that provide visitors (i.e., untrained non-staff members of the public) with the opportunity to have indirect and direct contact with live captive wild animals (both inside and outside of their permanent enclosures). For the purpose of this study, unlike Fernandez et al. [3] and their utilization of the term animal–visitor interaction, we did not include visitor presence (and associated observations) made from outside a captive wild animal enclosure alone as a type of AVI.

Modern zoos and aquaria worldwide already attract more than 700 million visits every year [1,11]. Given the recent and expected future global increases in wildlife tourism [10,12] there is a pressing need to audit the diversity of AVIs being offered in regard to their purpose and impact on wild animal welfare. Recent studies have reviewed the impacts of some AVIs offered by modern zoos and aquaria [3] and in other types of wildlife tourist attractions [10]. However, to date, no attempt has been made to describe the full diversity of AVIs currently being offered by modern zoos and aquaria. This type of information is of value for modern zoos and aquaria as it has the potential to inform both future research and operational initiatives required to better ensure that AVIs do not negatively impact on wild animal welfare (and instead provide either neutral or positive impacts) and to potentially enhance educational initiatives for visitors.

Here we attempt to describe the diversity of AVIs and their prevalence in zoos and aquaria. We asked: (1) What different types of AVIs are currently being advertised by zoos and aquaria, and what are the proportions of different types? (2) Which types of AVIs are most prevalent across different geographical regions? (3) Which taxonomic groups are most prevalent in AVIs? We believe the information gathered will help guide efforts to safeguard and improve the welfare of captive wild animals in modern zoos and aquaria.

## 2. Methods

### 2.1. Zoos and Aquaria Website Review

Founded in 1935 to help maximise their conservation impact, the World Association for Zoo and Aquariums (WAZA) considers itself to be the “unifying organisation for the world zoo and aquarium community” [13]. Currently, WAZA is comprised of approximately 282 direct institutional members and 22 different regional association members that together comprise approximately 1300 facilities globally [13]. We used predetermined criteria, applied by a single researcher to systematically search the official public websites of these 1300 facilities between November 2016 and March 2017 for information about the AVIs that they advertised. We only included facilities in our review if (1) it was part of a regional or national association that officially declared WAZA affiliation on its website and (2) possessed an official public website using text that could be accessed in English (either directly or by using Google Translate).

We gave each selected facility a unique identification code noting: (1) The website address; (2) the type of facility (“zoo”, “aquarium”, or “both”); (3) the type of WAZA affiliation (“direct WAZA membership”, “regional association membership”, or “both”); (4) the region and country of operation; and (5) the date of review (Supplementary Material File S1). We then screened facility webpages for evidence of any AVIs involving non-domesticated species (i.e., those that have not undergone significant genetic, behavioural and morphological changes from their wild ancestors, typically as a result of selective breeding [13]) by reading the text used to describe each facility and by examining associated images. We included any references to “camels” and “llamas” in our analyses as although they are most likely domesticated individuals (see Reference [14] for more details) we could not fully confirm their non-wild status from our desktop review alone. However, to ensure that this fact was not lost, these species were highlighted in relevant analyses. Where relevant information was available, we categorised advertised AVIs as either “direct” or “indirect”, depending on whether or not it was implied that visitors were permitted to have direct physical contact with the wild animals involved. We further categorised direct AVIs as: (1) “feeding”; (2) “petting”; (3) “riding”; and (4) “walk or swim with”, according to definitions provided in Table 1. We further categorised indirect AVIs as: (5) “non-hand feeding” (6) “walk through or swim through”; (7) “drive through or cage dive”; and (8) “show and performance”, according to definitions provided in Table 1.

Where relevant information was available, we also noted the taxonomic class, and the taxonomic order for any of the wild animal(s) involved. For vertebrates, we classified the wild animals involved as Actinopterygii (“bony” fish), Amphibia (amphibians), Aves (birds), Chondrichthyes (sharks and rays), Mammalia (mammals), or Reptilia (reptiles). We classified all other wild animals as invertebrates (i.e., Anthozoa (e.g., sea anemone and coral), Arachnida (spiders and scorpions), Cephalopoda (squid and octopus), Diplopoda (millipedes), Echinoidea (sea urchins), Gastropoda (snails and slugs), Holothuroidea (sea cucumbers), Insecta (insects), Malacostraca (crabs, lobsters and woodlice), Merostomata (horse shoe crabs), Polyplacophora (chitons), and Scyphozoa (jellyfish)). For all wild animals, we assigned taxonomic nomenclature according to IUCN Red List of Threatened Species (IUCN 2016).

### 2.2. Statistical Analyses

All statistical analyses were carried out using R statistical software version 3.4.1 (R Development Core Team, 2017, Vienna, Austria. A Kruskal–Wallis test was used to test for a difference in the number of AVI types per facility between regions and a pairwise Wilcoxon rank sum test with Holm-Bonferroni correction for multiple comparisons to test for differences between the regions. Chi-square goodness of fit test was used to investigate the distributions of AVI frequency across region with each AVI type analysed independently, with the null hypotheses that there was an even distribution weighted by the number of zoo surveyed in each region [15]. *p*-values were adjusted for multiple comparisons using the false discovery rate (fdr) correction [16]. For each AVI type, the frequency of the species class was explored separately and a goodness of fit chi-square test was also applied to test whether species class were used equally across each type of interaction. *p*-values were again adjusted for multiple comparisons using the false discovery rate (fdr) correction [16].

## 3. Results

**Figure 1:**
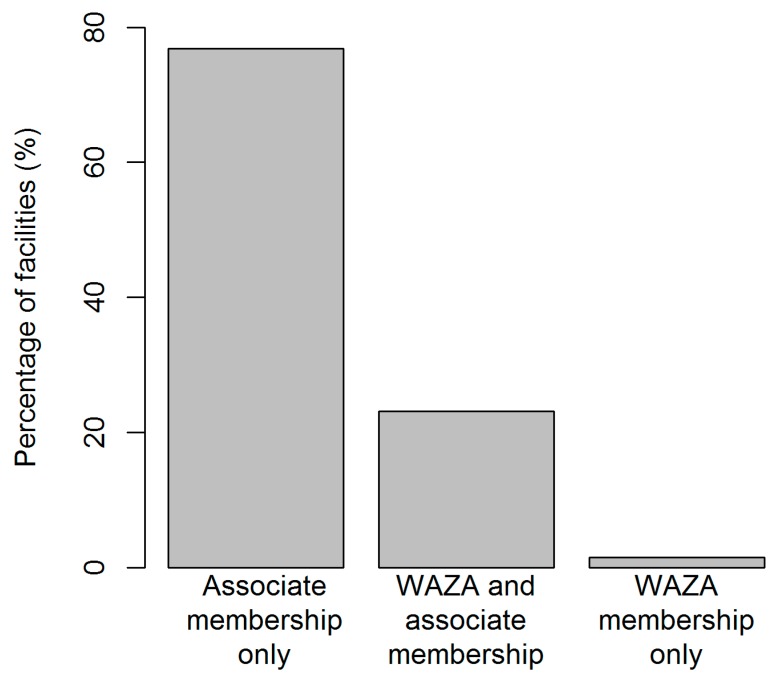
The number of facilities with a World Association for Zoos and Aquariums (WAZA) membership only, a WAZA and an association membership, and an association membership only.

We included a total of 1241 different facilities in our website review (Supplementary Material File S1). With regards to type, the majority of these facilities were zoos (*n* = 845; 68%) followed by those that included both zoo and aquarium components (20%), and aquaria (12%). With regards to affiliation, the majority of facilities (*n* = 940; 77%) were members of WAZA’s regional or national association members, followed by those that were both direct members of WAZA and other regional or national associations (23%) (Figure 1). Only 2% of the facilities included in this review were solely affiliated to WAZA directly (Figure 1). 

**Figure 2:**
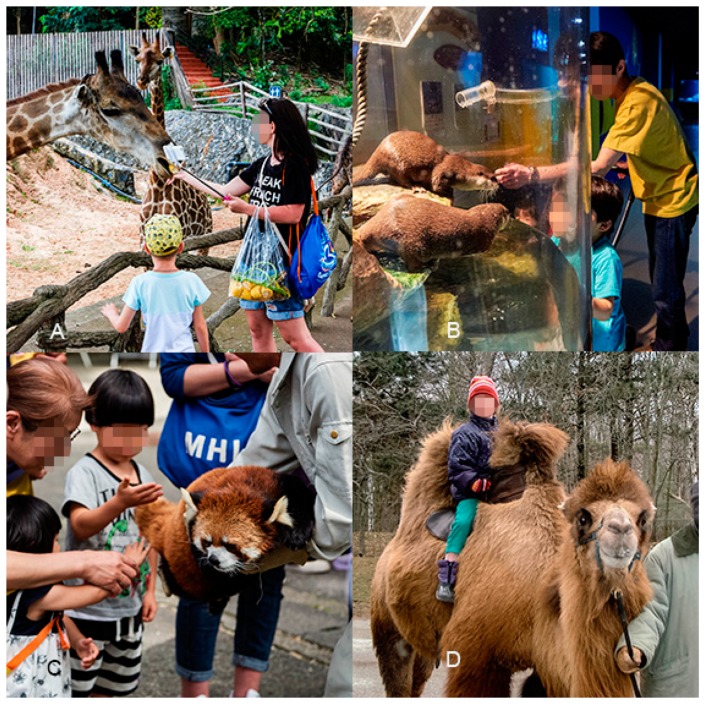
Example images for four of the different AVI types described in this study: (**A**) Hand feeding of a giraffe (*Giraffa* sp.); (**B**) Non-hand feeding of a small-clawed otter (*Aonyx cinereus*); (**C**) Petting of a red panda (*Ailurus fulgens*); (**D**) Riding of a Bactrian camel (*Camelus bactrianus*). Images A, B and C were taken by a field researcher during visits to facilities (copyright Fernando Carniel Machado); image D was obtained via Flickr (copyright Paul (CC BY-SA 2.0)). All images were taken at facilities that fall under the category of belonging to WAZA regional association members, they have been altered to ensure any individuals are made anonymous and are representative of screen shots included in the data set of this study.

**Figure 3:**
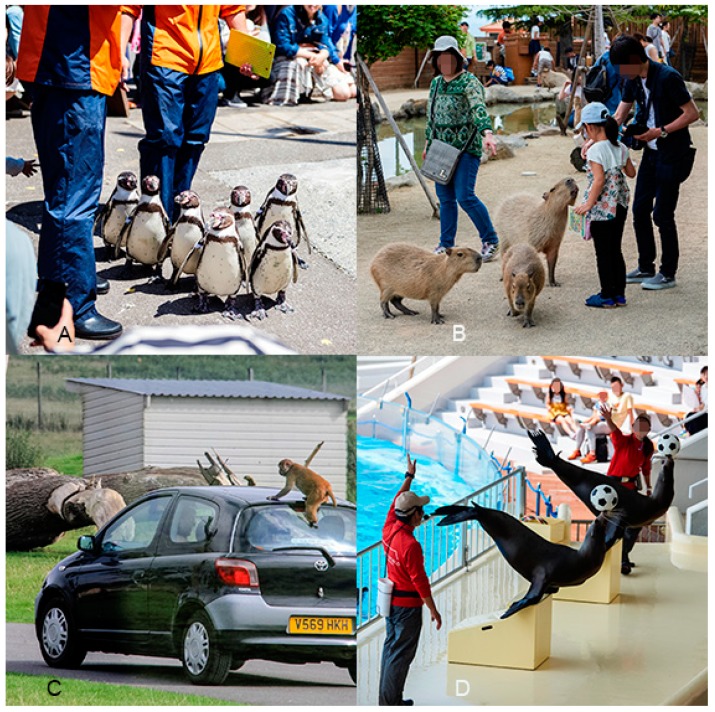
Example images for four different AVI types described in this study: (**A**) Walking with an African penguin (*Spheniscus demersus*); (**B**) Walking through a capybara enclosure (*Hydrochoerus hydrochaeris*); (**C**) Driving through a macaque enclosure (*Macaca* sp.); (**D**) Show and performance with sea lions (*Zalophus californianus*). Images were taken from screenshots of our data set included in this study. Images A, B and D were taken by a field researcher during visits to facilities (copyright Fernando Carniel Machado); image C was obtained via Flickr (copyright Karen Roe (CC BY-SA 2.0)). All images were taken at facilities that fall under the category of belonging to WAZA regional association members, they have been altered to ensure any individuals are made anonymous and are representative of screen shots included in the data set of this study.

**Figure 4:**
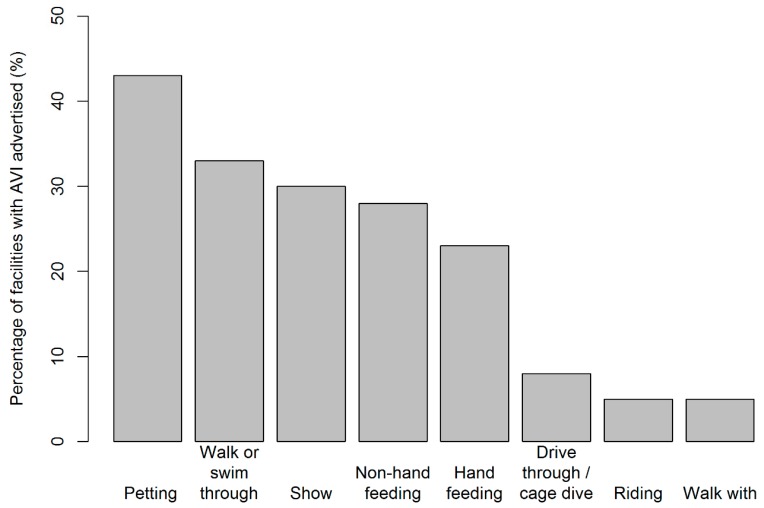
Percentage of facilities observed with each AVI type. Frequency of AVI type was found to be unevenly distributed across the facilities reviewed (χ^2^ = 830.56, df = 7, *p* < 0.001); with petting, walk or swim through, show, non-hand feeding, and hand feeding found to occur more frequently than expected.

Overall, at least one type of AVI was advertised by 929 (75%) of the facilities included in our review and 587 (47%) facilities advertised more than one type of AVI. Petting (*n* = 534; 43% of facilities) was the most common type of AVI observed, followed by walk through or swim through opportunities (33%), shows and performances (30%), non-hand feeding (28%), hand feeding (23%), drive through or cage dives (8%), riding (5%) and walk with opportunities (5%) (Figure 2, Figure 3 and Figure 4).

There was a significant association between the presence–absence of an AVI and membership category (χ^2^ = 25.3, df = 3, *p* < 0.001) with “affiliated membership only” (AO) facilities, “no membership” (NN) facilities and “WAZA only” (WO) facilities having a higher number of facilities without AVIs than expected, and “both WAZA and affiliated membership” (BA) facilities having a higher than expected number of facilities with AVI present. A goodness of fit chi-square test was then applied to each AVI type to see if the frequency of AVI was evenly distributed (weighted based on the number of facilities in each membership category). AVI types “handfeeding” (χ^2^ = 58.3, df = 3, *p* < 0.001), “ride” (χ^2^ = 9.1, df = 3, *p* = 0.044), “Walk or Swim with” (χ^2^ = 14, df = 3, *p* = 0.008), “drive through” (χ^2^ = 12.1, df = 3, *p* = 0.014) and “show” (χ^2^ = 15.4, df = 3, *p* = 0.006) were significantly higher than expected in facilities associated with both direct WAZA members and regional association members. *p*-values were adjusted for multiple comparisons.

### 3.1. Regional Variation in AVIs

**Figure 5:**
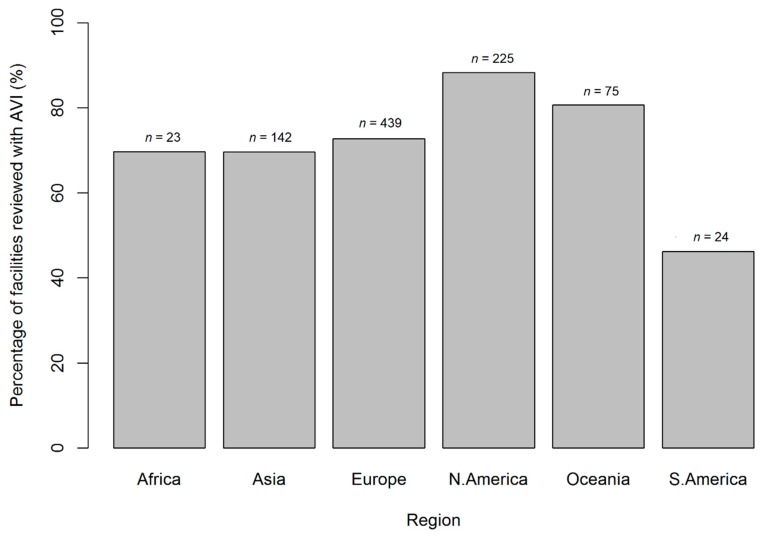
Percentage of facilities with an advertised AVI split by regions (number above each bar indicates the actual number of facilities with an AVI). A significant difference between the observed frequency of facilities with AVIs per region and those expected (by an even distribution weighted by the number of facilities surveyed per region (χ^2^ = 13.5, df = 5, *p* = 0.019)), with North America and Oceania having a higher frequency of AVIs than expected.

The average number of AVI types present per facility was 1.7 ± 1.6 (mean ± SD, median = 1) with a significant difference in the number of AVI types per facility between regions (Kruskal–Wallis χ^2^ = 101.7, df = 5, *p* < 0.001), with North America and Oceania having a significantly higher number of AVI types per facility then the other regions (Pairwise Wilcoxon rank sum test: *p* < 0.05). With regards to region of operation, the largest numbers of facilities reviewed were located in Europe (*n* = 604; 49%), followed by North America (21%), Asia (16%), Oceania (7%), South America (including Central America and the Caribbean) (4%), and Africa (including the Middle East) (3%). Europe and North America had the highest frequency of facilities with at least one AVI type in this study (*n* = 439, *n* = 225, respectively). However, North America and Oceania had the highest percentage of facilities surveyed with AVIs based on the presence or absence of at least one AVI type (Figure 5). There was a significant difference between the observed frequency of facilities with at least one AVI type per region and those expected (by an even distribution weighted by the number of facilities surveyed per region (χ^2^ = 13.5, df = 5, *p* = 0.019)), with North America and Oceania having a higher observed frequency of AVIs than expected (Figure 5).

**Figure 6:**
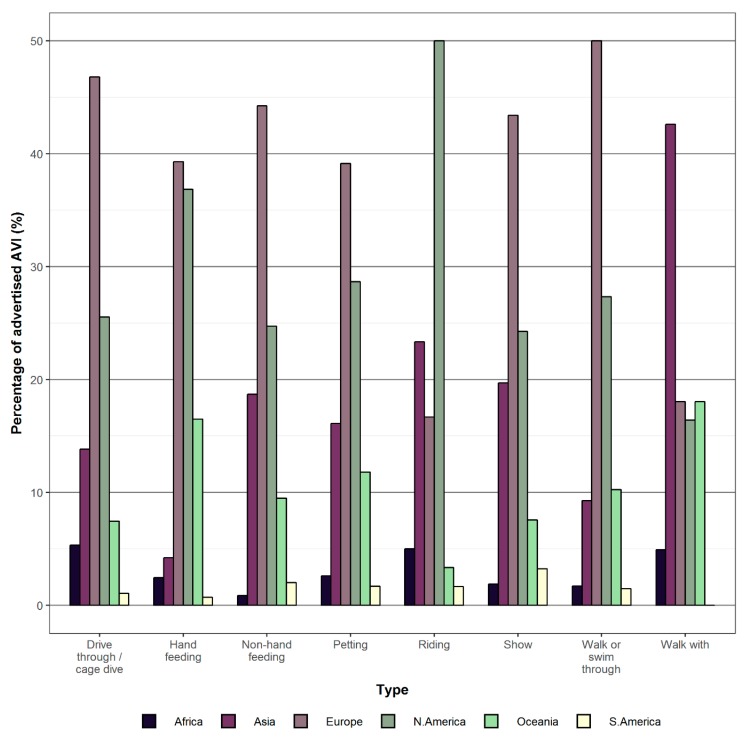
The percentage of advertised AVIs split by region and AVI type.

With regards to regional occurrence, the occurrence of hand feeding (χ^2^ = 107, df = 5, *p* < 0.001) and petting interactions (χ^2^ = 48.3, df = 5, *p* < 0.001) in North America and Oceania were significantly higher than expected. The occurrence of non-hand feeding AVIs were also significantly higher in North America, Oceania and in Asia (χ^2^ = 15.4, df = 5, *p* = 0.012). Ride interactions were significantly higher than expected in North America and Asia (χ^2^ = 43.2, df = 5, *p* < 0.001) and walk-with interactions were significantly higher in Asia, Oceania and Africa (χ^2^ = 50.5, df = 5, *p* < 0.001). Walk- or swim-through interactions were significantly more common than expected in North America, Oceania and Europe (χ^2^ = 34.9, df = 5, *p* < 0.001). Lastly, drive through (χ^2^ = 6.3, df = 5, *p* = 0.279) interactions and shows (χ^2^ = 8.6, df = 5, *p* = 0.143) were not significantly different than expected, weighted based on the number of facilities surveyed per region (Figure 6). *p*-values were adjusted for multiple comparisons.

### 3.2. Taxonomic Variation in AVIs

**Figure 7:**
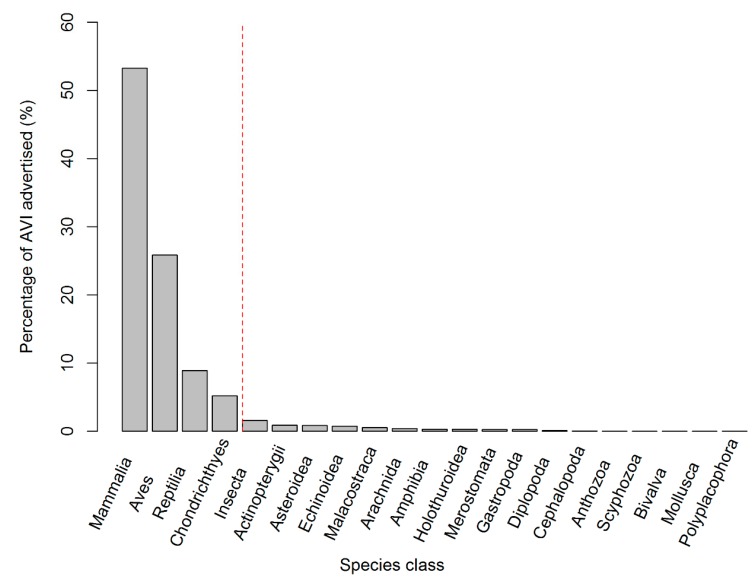
Percentage of advertised AVIs split by species class. A significant difference between the observed frequency of AVIs per species class and those expected by an even distribution (χ^2^ = 33,940, df = 20, *p* < 0.001), with Mammalia, Aves, Reptilia and Chondrichthyes having a higher AVI frequency than expected (left of the dashed red line).

A significant difference was found between the observed frequency of AVIs per species class and those expected by an even distribution (χ^2^ = 33,940, df = 20, *p* < 0.001), with Mammalia (*n* = 5142, 53% (2739)), Aves (26%), Reptilia (9%) and Chondrichthyes (5%) having a higher AVI frequency than expected (Figure 7). The frequency of AVI was also unevenly distributed across species order (χ^2^ = 26589, df = 89, *p* < 0.001), with 18 of the 90 orders observed being higher in frequency than expected and the top five orders accounting for over 50% of all the AVIs in this study (Carnivora (carnivores) = 18%; Artiodactyla (even-toed ungulates) = 13%; Primata (primates) = 8%; Psittaciformes (parrots) = 7%; and Squamata (lizards and snakes) = 5% (Supplementary Material Figure S1)).

**Figure 8:**
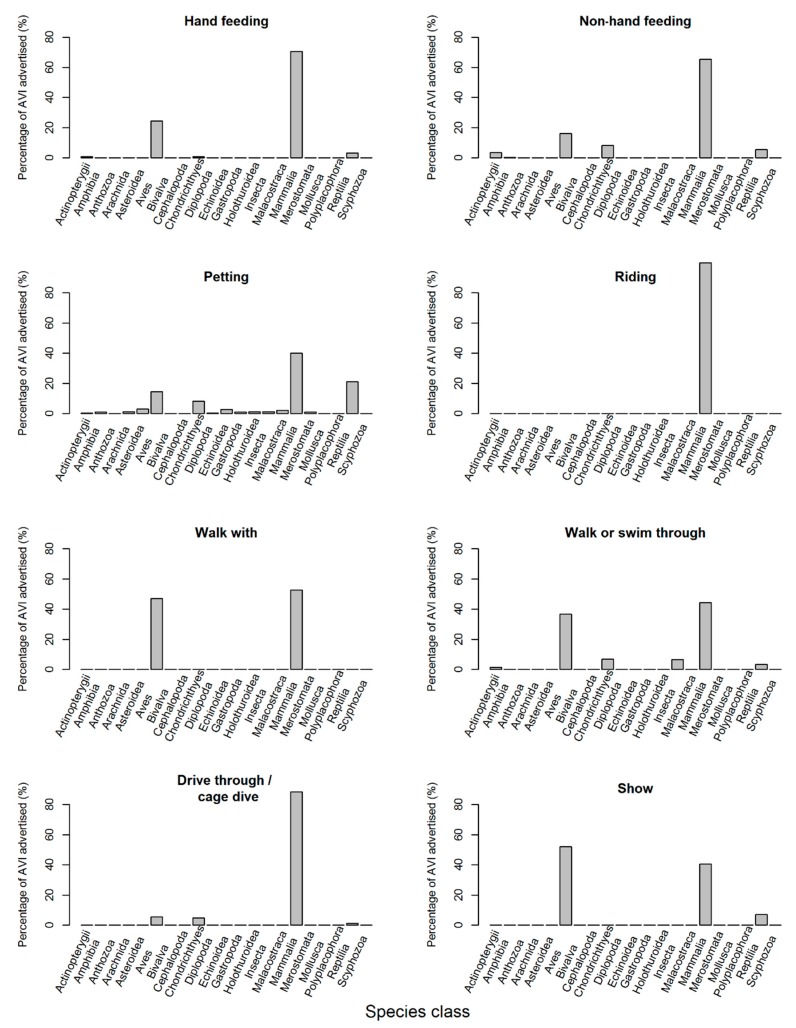
Percentage of advertised AVIs advertised per species class split by AVI type. A significant difference between the observed AVI frequency per species class and those expected with an even distribution were found for each AVI type (hand feeding: χ^2^ = 5845, df = 20, *p* < 0.001; non-hand feeding: χ^2^ = 4641, df = 20, *p* < 0.001; petting: χ^2^ = 5602, df = 20, *p* <0.001; riding: χ^2^ = 1340, df = 20, *p* < 0.001; walk with: χ^2^ = 686, df = 20, *p* < 0.001; walk or swim through: χ^2^ = 5953, df = 20, *p* < 0.001; drive through or cage dive: χ^2^ = 6810, df = 20, *p* < 0.001; show: χ^2^ = 6984, df = 20, *p* < 0.001). *p*-values were adjusted for multiple comparisons.

A significant difference between the observed AVI frequency per species class and those expected by an even distribution were found for each AVI type (*p* < 0.001, Figure 8). Mammalia, followed by Aves, were the most frequently occurring species class in hand feeding (Mammalia (71%); Aves; (24%)), non-hand feeding (Mammalia (63%); Aves (16%)); walk with (Mammalia (53%); Aves (47%)), walk or swim through (Mammalia (44%); Aves (37%)) and drive through or cage dive (Mammalia (89%); Aves (5%)). Mammalia were the most frequent class for petting followed by Reptilia (Mammalia (40%); Reptilia (21%)) and were the only class found in riding AVIs (Mammalia (100%)). Aves were the most frequently found class in shows followed by Mammalia (Aves (52%); Mammalia (41%)) (Figure 8).

At the order level, Artiodactyla were the most common order found in hand feeding (29%) (followed by Carnivora and Psittaciformes (15%, respectively)), riding (70%) (followed by Cetartiodactyla (odd-toed ungulates) (15%)) and drive through or cage dive type AVIs (44%) (followed by Carnivora (19%)). The most frequently occurring group for non-hand feeding and shows were Carnivora (34% and 26%, respectively) followed by Artiodactyla for non-hand feeding (10%) and Psittaciformes in shows (15%). Squamata and Carnivora were the most frequently advertised orders for petting AVIs (16% and 14%, respectively), Sphenisciformes (penguins) and Carnivora (39% and 36%, respectively) for walk with AVIs, and Primates and Diprotodontia (the largest marsupial order) (19% and 9%, respectively) for walk or swim through AVIs (Supplementary Material Table S1). Of these AVIs, 2% of the total were related to camels and llamas, which could not be determined as either wild or domesticated via the images provided on facility websites. These species accounted for 40% of all riding AVIs, 6% of walk with AVIs, 3% of non-hand feeding, 2% of drive through or cage dives, and 1% of both hand feeding and petting instances.

## 4. Discussion

We provide an initial insight into the extent, occurrence and characteristics of AVIs with wild animals taking place within zoos and aquaria across the globe. Our study revealed that a wide range of AVIs were being offered, ranging from those that involve no direct contact with people (e.g., drive through experiences), to those that involve repeated direct contact with multiple people including visitors and or staff (e.g., rides). Our study shows that certain AVI types (including drive through, hand feeding, riding, shows and performances, and walk with or swim with opportunities) were particularly prevalent in facilities with both direct WAZA membership and WAZA regional association membership. Overall, 75% of facilities included in our study offered some type of AVI, with petting captive wild animals being most prevalent across the globe.

From a regional perspective, our study shows that overall most AVI types follow similar levels of occurrence across all regions. However, there are certain AVI types that were more commonly offered than others. For example, opportunities to hand feed captive wild animals were particularly prevalent in North America and Oceania, opportunities for non-hand feeding were particularly prevalent in Asia, North America and Oceania, opportunities to “ride” wild animals were particularly prevalent in Asia and North America, opportunities to walk or swim through wild animal enclosures were particularly prevalent in Europe, North America and Oceania, and opportunities to “walk with” wild animals were particularly prevalent in Africa, Asia and Oceania. From a taxonomic perspective, our study shows that a wide range of different wild animals were involved with AVIs in zoos and aquaria, including at least 90 different taxonomic orders (Supplementary Material Table S1). However, overall, our study suggests that there was a significant preference for mammals in most AVIs. Furthermore, with regards to taxonomic orders, our study also suggests that there is an apparent preference for carnivores, even-toed ungulates, lizards, marsupials, odd-toed ungulates, parrots, penguins, primates and snakes.

Clearly, the nature of AVIs provided by zoos and aquaria varies widely, as do the taxa involved. Yet our knowledge of their impacts on these wild animals, including whether they are positive, neutral or negative, remains limited [6,7,11]. For example, to date, most research has focused more on the effects of visitor presence on captive wild animals rather than AVIs themselves (e.g., References [3,11,17,18,19]) or on selected taxonomic groups such as primates, felids and spheniscids [6,11]. Some research indicate that certain AVIs (when implemented according to best practice) can be rewarding and a positive source of enrichment for the wild animals involved (e.g., References [20,21]). Yet in contrast, other research indicate that certain AVIs can be a disturbing and negative source of distress for wild animals [22,23]. Brief summaries of research focused on AVI types highlighting the potential positive and negative impacts on animal welfare are summarised below.

## 5. The Potential Impacts of AVIs on Wild Animal Welfare

### 5.1. Direct and Indirect Feeding

There are some evidence that, in certain scenarios, this type of AVI can improve captive wild animal welfare as environmental enrichment and by alleviating unfulfilled foraging motivations. For example, Orban et al. [24] reported that certain individual giraffes involved in visitor feeding programs tended to perform less oral stereotypic behaviour such as object licking and tongue rolling. However, studies focused on this type of AVI in zoos and aquaria are limited with the potential negative impacts (e.g., over-feeding, ingestion of toxic substances, increased aggression, disrupted natural feeding behaviours and unintentional positive reinforcement of anticipatory or stereotypic behaviours) on captive wild animal welfare remaining largely unknown.

### 5.2. Petting and Walk with Interactions

Following a study focused on direct and close contact between common marmosets (*Callithrix jacchus*) and a familiar caretaker, Reference [20] suggested that programs involving simple unstructured interactions with humans could help maximise the welfare of captive nonhuman primates. However, other studies have reported on the potential negative welfare impacts that can be associated with this type of AVI. For example, Dans et al. [22] found that direct and close contact with American sea lions resulted in agonistic behaviour towards people such as bites. Similarly, Hogan et al. [25] found that this type of AVI lowered reactivity and avoidance of visitors but did not reduce the associated stress response of common wombat (*Vombatus ursinus*); suggesting that these animals entered into a state of “learned hopelessness”.

Animal interactions such as petting or “walk-with” experiences, require the animal to be trained so that it is safe to handle and responds positively towards the interaction. While positive interactions between humans and animals are important in that they influence animal welfare and can desensitise animals to some events and procedures and hence reduce any associated stress [26], training using techniques that create fear, pain and distress produce negative human–animal relationships and compromise animal welfare. Hand-reared animals may be used in animal interactions as they are more easily handled [26]. However, hand-reared animals can develop abnormal behaviours including stereotypies [26], indicating a negative impact on welfare.

No attempt to gather information on zoonotic disease prevention by modern zoos and aquaria (which is undoubtedly carried out by some facilities) was carried out as it was outside the scope of this study. However, wherever there is close or direct contact between animals and humans there is the risk of zoonotic disease transmission from the animals to humans and vice versa. For example, *Mycobacterium tuberculosis* (TB) may be transmitted between elephants and people [26], *Campylobacter* spp. can be transmitted between humans, livestock, domestic pets as well as zoo petting animals [27].

### 5.3. Walk and Swim Through Interactions

Jones et al. [28] and Collins et al. [29] found that crown lemurs (*Eulemur coronatus*) and ring-tailed lemurs (*Lemur catta*) adapted well and became habituated to visitor presence in their free ranging exhibits. Similarly, Martin and Melfi [30] reported that unfamiliar keeper presence did not appear to have detrimental effect on captive wild animal welfare for a number of species including slender tailed meerkat (*Suricata suricatta*). However, concerns remain that this type of AVI may be having negative impacts in certain scenarios. For example, those involving timid individuals and or species that are unable to adequately avoid unfamiliar visitors, sights and sounds [31]. Animals used for offering rides have little choice or control over their environment or activities, although providing choice such as refuge areas in swim with programmes could help increase control over the environment and reduce the likelihood of learned helplessness in captivity [26].

### 5.4. Drive Through and Cage Dive Interactions

Scientific studies focused on the welfare of captive wild animals involved in this type of AVI appear to be particularly lacking. Similar to walk and swim though attractions, it is assumed that some species are somewhat adaptable to this type of AVI. However, research focused on wild animals in their natural habitat has raised some concerns. For example, Huveneers et al. [32] found that cage diving can influence the fine-scale horizontal and vertical distribution, and rate of movement, of great white sharks (*Carcharodon carcharias*). They also suggested that this could lead to negative physiological impacts and potentially decrease individual or population fitness if elicited frequently or repetitively, or within important habitats (e.g., breeding or foraging areas).

### 5.5. Rides and Public Performances

Scientific studies focused on the animal welfare impacts of rides and public demonstrations involving captive wild animals also appear to be particularly lacking. Training, based on reward (positive reinforcement), may enhance health and reproductive potential of captive animals [33]. Wierucka et al. [21] noted general good health, an absence of stereotypic behaviours, and the presence of diversified natural behaviours with regards to cape fur seals (*Arctocephalus pusillus*) that were trained to entertain visitors. However, there are concerns that in some scenarios, training by means of negative reinforcement and punishment (which involves the use of painful stimuli) is used in order to train the wild animals to perform [34]. Some of these interactions may also require the animal to be restrained.

### 5.6. Recommendations to Help Safeguard and Improve Animal Welfare

Given the current prevalence and diversity of AVIs in zoos and aquaria, more research is required to investigate the impacts of AVIs on captive wild animal welfare. Our study provides a useful insight in the different types of AVI and taxonomic groups utilised by facilities (both globally and in different geographical regions) that can help to guide such future research effort and we believe would be a useful exercise to repeat in future as a source of comparison. However, it is important to note that impact of AVIs (both positive and negative) can vary greatly, not only depending on the biology and behaviour of a particular species and or individual wild animal, but also depending on other management practices employed at a given facility (for example, the frequency, duration and timing of an interaction, any associated animal training methods and the husbandry and housing provided, both during and after the interaction is concluded [6,11]). As such, we support the existing recommendation that all AVIs need to be assessed by applying relevant and ongoing welfare assessment and monitoring [6,11].

It has been suggested that the prevalence and diversity of AVIs in zoos and aquaria has largely been driven by the assumption that close proximity between animals and visitors, and where possible “actual contact”, can increase the likelihood, scope and impact of environmental education and public commitment to conservation actions [4,11]. However, research on this proposition has not yet produced definitive results [11]. There are also concerns that, in certain scenarios, AVIs may promote harmful attitudes and behaviours that impede animal welfare and conservation efforts [4]. For example, Ross et al. [35] found that when people viewed a photograph of a chimpanzee with a person close by, 35.5% incorrectly assumed chimpanzees were not an endangered species. Furthermore, people viewing the photographs considered chimpanzees to be an appealing pet. As such, we support the recommendation that zoos and aquaria should aim to explain the animal welfare and management processes to visitors taking part in AVIs [11]. We also recommend that increased research effort is required to establish the impact of AVIs on visitor attitudes and behaviour.

**Figure 9:**
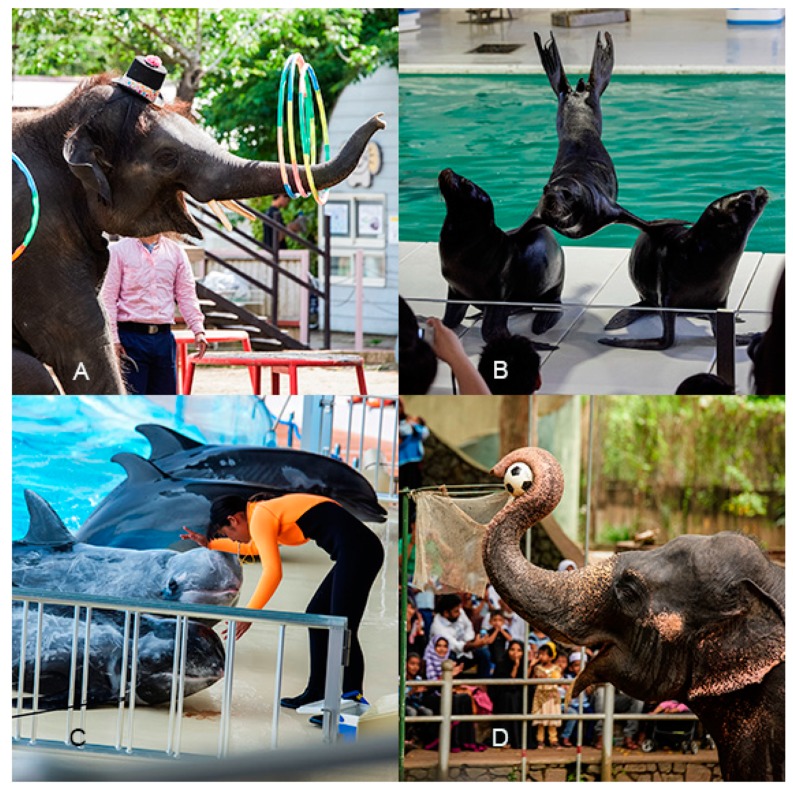
Example images for the four AVIs advertised that appear to be in contravention of WAZA’s animal welfare strategy. (**A**) An Asian elephant (*Elephas maximus*) in a top hat spinning “hoola” hoops for a show; (**B**) sea lions (*Zalophus californianus*) perform a “hand stand pyramid” for a show; (**C**) Dolphins (Delphinoidea spp.) beached for a show; and (**D**) an Asian elephant plays basketball for a show. Images were taken from screenshots of our data set included in this study. Images A, B, C and D were taken by a field researcher during visits to facilities (copyright Fernando Carniel Machado). Images A, C and D were taken at facilities that fall under the category of WAZA regional associate members. Image D was taken at a facility that falls under the category of being a direct member of WAZA. Images have been altered to ensure any individuals are made anonymous and are representative of screen shots included in the data set of this study.

It is likely that the lack of existing research has contributed to a lack of clarity regarding AVI practices that should be promoted or prohibited within zoos and aquaria. For example, WAZA recommends that its member organisations should not undertake, contribute or participate in animal shows, displays or interactive experiences where animals perform unnatural behaviours [11]. However, clear and precise definitions and guidelines for what does (and does not) constitute unnatural wild animal behaviour are currently lacking and can be considered ambiguous. Similarly, whether or not an AVI is treating the animals involved “with respect” [11] can also be highly subjective, culturally sensitive, and difficult to evaluate. This is exemplified by a number of AVIs documented from facilities (including those that are direct members of WAZA) that could appear to be in contravention of these recommendations, including elephant rides, photo opportunities with wild animals in costumes, and circus-like public demonstrations (Figure 9). As such, we recommend that more clarification should be provided to help guide best practice at facilities in this regard.

Currently, there is no global body regulating wildlife tourism, a situation that risks enabling visitor revenue to become the ultimate arbiter of what constitutes an acceptable AVI [12]. However, visitors are not adequate assessors of animal welfare in this context as they typically lack the specialist knowledge required and are subject to a number of psychological biases that obscure the ethical dimensions of decisions relating to AVI participation [12]. In the context of modern zoos and aquaria, a visitor’s ethical decision-making process is further complicated by the fact that operational standards, including those pertaining to AVIs, vary in description and application between global, regional and national zoological associations, national legislative standards and also between individual facilities. Furthermore, zoological associations do not all function in the same way. For example, some zoological associations (e.g., the European Association of Zoo and Aquaria (EAZA)) act as “accreditation” bodies, which set official standards and procedures that must be adhered to in order for affiliation to be retained (EAZA, 2018). However, others (e.g., WAZA) consider themselves more as “membership” bodies that provide guidance that does not necessarily need to be followed [11]. We suggest that more consistent guidance, adherence to agreed principles and more potent operating models would greatly aid existing and future efforts to safeguard animal welfare in this regard.

## 6. Limitations

Our study was necessarily descriptive and could not be exhaustive. Specifically, we restricted our analyses to a particular set of AVIs advertised on zoo and aquarium websites; and so those without an online presence do not feature in our findings. Equally, we recognise that information regarding the factors responsible for motivating zoos and aquaria to provide content on AVIs online (e.g., the influence of species rarity and charisma [12] or perceived public entertainment value) and how this differs across certain demographic groups (e.g., between countries and regions) is also lacking.

We also recognise that AVIs advertised online do not represent a full or unbiased depiction of these activities for a number of reasons, including the fact that they (and any associated content provided by facilities) do not represent a random sample, may be out of date (i.e., feature AVIs that have been adapted or are no longer practiced), and are liable to a degree of misinterpretation given the limited amount of information available. Furthermore, tourist facilities are not always accurate, or honest, when describing their AVIs to the public [12].

Independent ground level AVI audits using direct observations and interviews with staff focused on animal welfare aspects would be required to make a full and unbiased assessment of their impact. For example, the higher-than-expected advertisement of riding in Europe and USA may involve the involvement of domesticated camelids rather than their wild counterparts. In addition, a full review of existing literature focused on the animal welfare impacts of AVIs would be beneficial in this regard. Similarly, in order to carry out this study we were required to create arbitrary categorical classification system for AVIs advertised by modern zoos and aquaria, which has a direct influence on subsequent statistical analyses. Although we believe that our classification is both sensible and useful starting point in this regard, information gathered from future research may help to refine it.

Unfortunately, these independent ground level AVI audits are beyond the scope of this present study. However, in lieu of such readily accessible public information, and given the recognised marketing value of providing online information about specific activities to potential visitors before they attend a tourist attraction [36], we believe that this study of over 1000 websites represents one of the most comprehensive reviews focused on the diversity of AVIs and their prevalence in zoos and aquaria that has been carried out to date.

## 7. Conclusions

It is clear that a majority of zoos and aquaria are providing AVIs, presumably to help support their broad remit composed of animal welfare, conservation, education and research goals. Our preliminary review of existing research and WAZA’s animal welfare strategy suggests that, when implemented in a manner that prioritises animal welfare, certain AVIs can be rewarding and a positive source of enrichment for the wild animals involved [11]. Conversely, if poorly implemented, certain AVIs can compromise wild animal welfare. In light of the vast diversity of AVIs highlighted in our study, the research required to ensure that they are safe, non-stressful and provide positive experiences for the wild animals involved is currently lacking. As such, in addition to increased research effort focused on the potential impacts of AVIs on wild animal welfare and visitor behaviour, we support the recommendation that all AVIs should be independently assessed and monitored against a measurable standard as part of an on-going accreditation programme, on an on-going basis, by experienced captive wild animal welfare professionals. Taken together, the resulting information can help to identify practices that should be encouraged to help improve captive wild animal welfare, and to identify those practices that should be prohibited in modern zoos and aquaria. We hope that our global review will prove valuable to modern zoos and aquaria facilities that wish to operate in a manner that prioritises animal welfare. Specifically, it identifies the main different AVI types currently being advertised by zoos and aquaria, which types are most prevalent across different geographic regions and which taxonomic groups are most prevalent in AVIs. Such information has the potential inform both future research and operational animal welfare focused initiatives and to guide the most effective allocation of resources in this regard.

## Figures and Tables

**Table 1:** Animal–visitor interaction (AVI) definition criteria used during this study.

Number	AVI Type	Contact Type	Definition Criteria
1	Hand Feeding	Direct	Interactions where visitors can enter into close proximity to a captive wild animal, and provide food and water by hand, with or without a physical barrier between them, with or without official staff supervision. Visitors are likely to have a relatively high expectation of direct contact.
2	Non-Hand Feeding	Indirect	Interactions where visitors can enter into close proximity with a captive wild animal and provide food and water, although not by hand, with or without a physical barrier between them, with or without official staff supervision. Visitors are likely to have a relatively low expectation of direct contact.
3	Petting	Direct	Interactions where visitors can enter into close proximity with a captive wild animal to hold and touch them, with or without any physical barrier between them, with or without official staff supervision. Visitors are likely to have a relatively high expectation of direct contact.
4	Riding	Direct	Interactions where visitors can enter into close proximity with a captive wild animal, which will carry them whilst in motion, with or without a harness or equivalent, with or without official staff supervision. Visitors are likely to have a relatively high expectation of direct contact.
5	Walk with or Swim with	Direct or Indirect	Interactions where visitors can experience close proximity to a captive wild animal, which is typically restrained by a harness or equivalent, without any physical barrier, with or without official staff supervision. Visitors are likely to have a relatively moderate expectation of direct contact.
6	Walk or Swim Through	Indirect	Interaction where visitors can experience close proximity to a captive wild animal without any physical barrier, with or without official staff supervision. Visitors are likely to have a relatively low expectation of direct contact.
7	Drive through or Cage dive	Indirect	Interactions where visitors can experience close proximity to a captive wild animal with a vehicle or device acting as a physical barrier, with or without official staff supervision. Visitors are likely to have a relatively low expectation of direct contact.
8	Show and Performance	Indirect	Interactions with trained staff and or visitors where a captive wild animal, provides a demonstration of either natural or non-natural behaviour for visitors, with or without a physical barrier between them, under official staff supervision. Visitors are likely to have a relatively low expectation of direct contact.

## Supplementary Materials

The following are available online at https://www.mdpi.com/2076-2615/9/6/332/s1. File S1: ZOOvai.data1, Figure S1: Percentage advertised AVIs split by species order. Orders left of the red dashed line were higher in frequency than expected given an even distribution, Table S1: Percentage advertised AVIs per species order split by AVI type.
