# Supplementary material for: A Global Review of Animal–Visitor Interactions in Modern Zoos and Aquariums and Their Implications for Wild Animal Welfare
Source: Animals (Basel). 2019 Jun 8;9(6):332. doi: 10.3390/ani9060332 (PMC6617332; doi:10.3390/ani9060332)
Supplement: Supplementary file 1 [file animals-09-00332-s001.zip › animals-500636-supplementary-Final.docx]

Article

**A Global Review of Animal–Visitor Interactions in Modern Zoos and Aquariums and Their Implications for Wild Animal Welfare**

**Neil D’Cruze Sophie Khan, Gemma Carder, David Megson, Emma Coulthard, John Norrey and Georgina Groves**

**Supplementary Materials**

**Table S1.** Percentage advertised AVIs per species order split by AVI type.

| **Order** | **AVI Type** | | | | | | | |
| --- | --- | --- | --- | --- | --- | --- | --- | --- |
|  | **Hand feeding** | **Non-hand feeding** | **Petting** | **Riding** | **Walk with** | **Walk or swim through** | **Drive through or cage dive** | **Show** |
| Accipitriformes | 0.0 | 0.2 | 0.7 | 0.0 | 2.9 | 0.6 | 0.0 | 7.3 |
| Acipenseriformes | 0.0 | 0.0 | 0.0 | 0.0 | 0.0 | 0.1 | 0.0 | 0.0 |
| Actinaria | 0.0 | 0.0 | 0.2 | 0.0 | 0.0 | 0.0 | 0.0 | 0.0 |
| Afrosoricida | 0.0 | 0.0 | 0.2 | 0.0 | 0.0 | 0.1 | 0.0 | 0.0 |
| Anguilliformes | 0.2 | 0.4 | 0.0 | 0.0 | 0.0 | 0.2 | 0.0 | 0.0 |
| Anseriformes | 1.7 | 1.0 | 0.1 | 0.0 | 0.0 | 1.9 | 0.0 | 0.4 |
| Anura | 0.0 | 0.4 | 0.8 | 0.0 | 0.0 | 0.1 | 0.0 | 0.0 |
| Araneae | 0.0 | 0.0 | 1.4 | 0.0 | 0.0 | 0.1 | 0.0 | 0.1 |
| Artiodactyla | 29.3 | 10.4 | 4.6 | 70.1 | 7.1 | 6.3 | 44.1 | 0.4 |
| Aspidochirotida | 0.0 | 0.0 | 0.1 | 0.0 | 0.0 | 0.0 | 0.0 | 0.0 |
| Bivalvia | 0.0 | 0.0 | 0.1 | 0.0 | 0.0 | 0.0 | 0.0 | 0.0 |
| Blattodea | 0.0 | 0.2 | 0.5 | 0.0 | 0.0 | 0.2 | 0.0 | 0.0 |
| Bucerotiformes | 0.0 | 0.4 | 0.1 | 0.0 | 0.0 | 0.6 | 0.2 | 1.1 |
| Caenogastropoda | 0.0 | 0.0 | 0.1 | 0.0 | 0.0 | 0.0 | 0.0 | 0.0 |
| Camarodonta | 0.0 | 0.0 | 0.2 | 0.0 | 0.0 | 0.0 | 0.0 | 0.0 |
| Caprimulgiformes | 0.0 | 0.4 | 0.0 | 0.0 | 0.0 | 0.2 | 0.0 | 0.1 |
| Carcharhiniformes | 0.0 | 0.2 | 0.3 | 0.0 | 0.0 | 0.8 | 1.2 | 0.0 |
| Cariamiformes | 0.0 | 0.0 | 0.0 | 0.0 | 0.0 | 0.0 | 0.0 | 0.6 |
| Carnivora | 14.8 | 34.7 | 13.6 | 4.5 | 35.7 | 6.4 | 19.3 | 26.4 |
| Casuariiformes | 0.0 | 0.0 | 0.1 | 0.0 | 0.0 | 0.1 | 0.2 | 0.0 |
| Cathartiformes | 0.0 | 0.4 | 0.2 | 0.0 | 1.4 | 0.3 | 0.0 | 3.3 |
| Certartiodactyla | 1.5 | 0.2 | 0.6 | 0.0 | 2.9 | 0.1 | 3.7 | 0.0 |
| Cetacea | 0.9 | 1.0 | 5.5 | 14.9 | 0.0 | 1.6 | 0.0 | 14.4 |
| Characiformes | 0.4 | 0.4 | 0.0 | 0.0 | 0.0 | 0.0 | 0.0 | 0.0 |
| Charadriiformes | 0.2 | 0.0 | 0.1 | 0.0 | 0.0 | 0.9 | 0.0 | 0.1 |
| Chiroptera | 0.4 | 1.0 | 0.0 | 0.0 | 0.0 | 1.2 | 0.0 | 0.1 |
| Ciconiiformes | 0.0 | 0.4 | 0.0 | 0.0 | 0.0 | 1.2 | 0.0 | 1.0 |
| Cingulata | 0.0 | 0.0 | 1.1 | 0.0 | 0.0 | 0.6 | 0.0 | 0.3 |
| Colliiformes | 0.0 | 0.0 | 0.0 | 0.0 | 0.0 | 0.1 | 0.0 | 0.0 |
| Columbiformes | 0.2 | 0.0 | 0.2 | 0.0 | 0.0 | 1.1 | 0.0 | 0.4 |
| Coraciiformes | 0.0 | 0.0 | 0.0 | 0.0 | 0.0 | 0.8 | 0.0 | 0.6 |
| Crocodilia | 0.0 | 1.2 | 2.4 | 0.0 | 0.0 | 0.1 | 0.5 | 2.4 |
| Cuculiformes | 0.0 | 0.0 | 0.0 | 0.0 | 0.0 | 0.1 | 0.0 | 0.1 |
| Cypriniformes | 0.2 | 1.4 | 0.4 | 0.0 | 0.0 | 0.0 | 0.0 | 0.0 |
| Dasyuromorphia | 0.0 | 0.2 | 0.2 | 0.0 | 0.0 | 0.0 | 0.0 | 0.0 |
| Decapoda | 0.0 | 0.0 | 2.4 | 0.0 | 0.0 | 0.0 | 0.0 | 0.0 |
| Didelphimorphia | 0.0 | 0.0 | 0.2 | 0.0 | 0.0 | 0.2 | 0.0 | 0.1 |
| Diprotodontia | 7.6 | 2.4 | 5.6 | 0.0 | 1.4 | 8.7 | 0.2 | 0.4 |
| Eulipotyphla | 0.0 | 0.0 | 0.5 | 0.0 | 0.0 | 0.0 | 0.0 | 0.3 |
| Eurypygiformes | 0.0 | 0.0 | 0.0 | 0.0 | 0.0 | 0.1 | 0.0 | 0.0 |
| Falconiformes | 0.0 | 0.0 | 0.2 | 0.0 | 0.0 | 0.1 | 0.0 | 2.2 |
| Forcipulatida | 0.0 | 0.0 | 0.1 | 0.0 | 0.0 | 0.0 | 0.0 | 0.0 |
| Galliformes | 0.2 | 0.2 | 0.0 | 0.0 | 0.0 | 2.0 | 0.0 | 0.3 |
| Gruiformes | 0.0 | 0.0 | 0.0 | 0.0 | 0.0 | 1.0 | 0.2 | 1.1 |
| Heterodontiformes | 0.0 | 0.0 | 0.0 | 0.0 | 0.0 | 0.1 | 0.0 | 0.0 |
| Hexanchiformes | 0.0 | 0.0 | 0.0 | 0.0 | 0.0 | 0.1 | 0.2 | 0.0 |
| Lamniformes | 0.0 | 0.0 | 0.0 | 0.0 | 0.0 | 1.0 | 0.9 | 0.0 |
| Lepidoptera | 0.0 | 0.0 | 0.0 | 0.0 | 0.0 | 7.1 | 0.0 | 0.0 |
| Monotremata | 0.2 | 0.0 | 0.4 | 0.0 | 0.0 | 0.0 | 0.0 | 0.1 |
| Musophagiformes | 0.0 | 0.0 | 0.0 | 0.0 | 0.0 | 0.9 | 0.0 | 0.0 |
| Myliobatiformes | 0.7 | 5.3 | 5.6 | 0.0 | 0.0 | 2.1 | 0.5 | 0.0 |
| Neoloricata | 0.0 | 0.0 | 0.1 | 0.0 | 0.0 | 0.0 | 0.0 | 0.0 |
| Octopoda | 0.0 | 0.0 | 0.2 | 0.0 | 0.0 | 0.0 | 0.0 | 0.0 |
| Orectolobiformes | 0.0 | 0.4 | 0.9 | 0.0 | 0.0 | 0.7 | 0.5 | 0.0 |
| Osteoglossiformes | 0.0 | 0.2 | 0.0 | 0.0 | 0.0 | 0.0 | 0.0 | 0.0 |
| Otidiformes | 0.0 | 0.0 | 0.0 | 0.0 | 0.0 | 0.1 | 0.0 | 0.0 |
| Passeriformes | 0.0 | 0.0 | 0.1 | 0.0 | 0.0 | 2.4 | 0.0 | 0.3 |
| Pelecaniformes | 0.2 | 2.4 | 0.1 | 0.0 | 1.4 | 4.4 | 0.5 | 1.3 |
| Perciformes | 0.0 | 1.2 | 0.0 | 0.0 | 0.0 | 1.1 | 0.0 | 0.0 |
| Perissodactyla | 3.1 | 3.3 | 3.7 | 0.0 | 0.0 | 0.2 | 18.4 | 0.1 |
| Phasmida | 0.0 | 0.0 | 0.5 | 0.0 | 0.0 | 0.0 | 0.0 | 0.0 |
| Phoenicopteriformes | 0.4 | 0.8 | 0.1 | 0.0 | 0.0 | 1.2 | 0.5 | 0.4 |
| Piciformes | 0.0 | 0.2 | 0.2 | 0.0 | 0.0 | 0.6 | 0.0 | 0.3 |
| Pilosa | 0.2 | 1.2 | 0.7 | 0.0 | 0.0 | 1.4 | 0.0 | 0.4 |
| Pinnipedia | 0.0 | 0.2 | 0.4 | 0.0 | 0.0 | 0.0 | 0.0 | 1.0 |
| Pleuronectiformes | 0.0 | 0.0 | 0.0 | 0.0 | 0.0 | 0.1 | 0.0 | 0.0 |
| Primates | 9.8 | 9.6 | 5.4 | 0.0 | 0.0 | 18.7 | 2.1 | 1.3 |
| Proboscidea | 2.4 | 2.2 | 1.9 | 10.4 | 5.7 | 0.2 | 1.6 | 1.5 |
| Psittaciformes | 14.6 | 1.2 | 5.6 | 0.0 | 0.0 | 8.5 | 0.0 | 14.5 |
| Rajiformes | 0.0 | 0.2 | 0.1 | 0.0 | 0.0 | 0.1 | 0.0 | 0.0 |
| Rhinopristiformes | 0.0 | 0.2 | 0.0 | 0.0 | 0.0 | 0.2 | 0.0 | 0.0 |
| Rhynchocephalia | 0.0 | 0.2 | 0.2 | 0.0 | 0.0 | 0.0 | 0.0 | 0.1 |
| Rodentia | 0.7 | 1.6 | 1.4 | 0.0 | 1.4 | 1.7 | 0.2 | 0.7 |
| Scorpiones | 0.0 | 0.0 | 0.1 | 0.0 | 0.0 | 0.1 | 0.0 | 0.0 |
| Semaeostomeae | 0.0 | 0.0 | 0.2 | 0.0 | 0.0 | 0.0 | 0.0 | 0.0 |
| Sirenia | 0.0 | 0.0 | 0.2 | 0.0 | 0.0 | 0.0 | 0.0 | 0.1 |
| Sorbeoconcha | 0.0 | 0.0 | 0.2 | 0.0 | 0.0 | 0.0 | 0.0 | 0.0 |
| Sphenisciformes | 5.2 | 6.5 | 2.9 | 0.0 | 38.6 | 3.2 | 0.0 | 0.6 |
| Squaliformes | 0.0 | 0.0 | 0.1 | 0.0 | 0.0 | 0.0 | 0.0 | 0.0 |
| Squamata | 0.4 | 0.8 | 15.5 | 0.0 | 0.0 | 1.5 | 0.0 | 5.3 |
| Squatiniformes | 0.0 | 0.0 | 0.0 | 0.0 | 0.0 | 0.1 | 0.2 | 0.0 |
| Strigiformes | 0.0 | 0.6 | 2.4 | 0.0 | 1.4 | 0.6 | 0.0 | 7.0 |
| Struthioniformes | 1.8 | 1.2 | 0.3 | 0.0 | 0.0 | 0.9 | 4.0 | 0.3 |
| Stylommatophora | 0.0 | 0.0 | 0.2 | 0.0 | 0.0 | 0.0 | 0.0 | 0.0 |
| Suliformes | 0.0 | 0.0 | 0.0 | 0.0 | 0.0 | 0.0 | 0.0 | 0.3 |
| Syngnathiformes | 0.0 | 0.2 | 0.1 | 0.0 | 0.0 | 0.0 | 0.0 | 0.0 |
| Testudines | 3.0 | 3.5 | 6.4 | 0.0 | 0.0 | 2.1 | 0.7 | 0.7 |
| Tubulidentata | 0.0 | 0.0 | 0.3 | 0.0 | 0.0 | 0.2 | 0.0 | 0.1 |
| Urodela | 0.0 | 0.0 | 0.1 | 0.0 | 0.0 | 0.0 | 0.0 | 0.0 |
| Xiphosura | 0.0 | 0.2 | 1.2 | 0.0 | 0.0 | 0.0 | 0.0 | 0.0 |

**
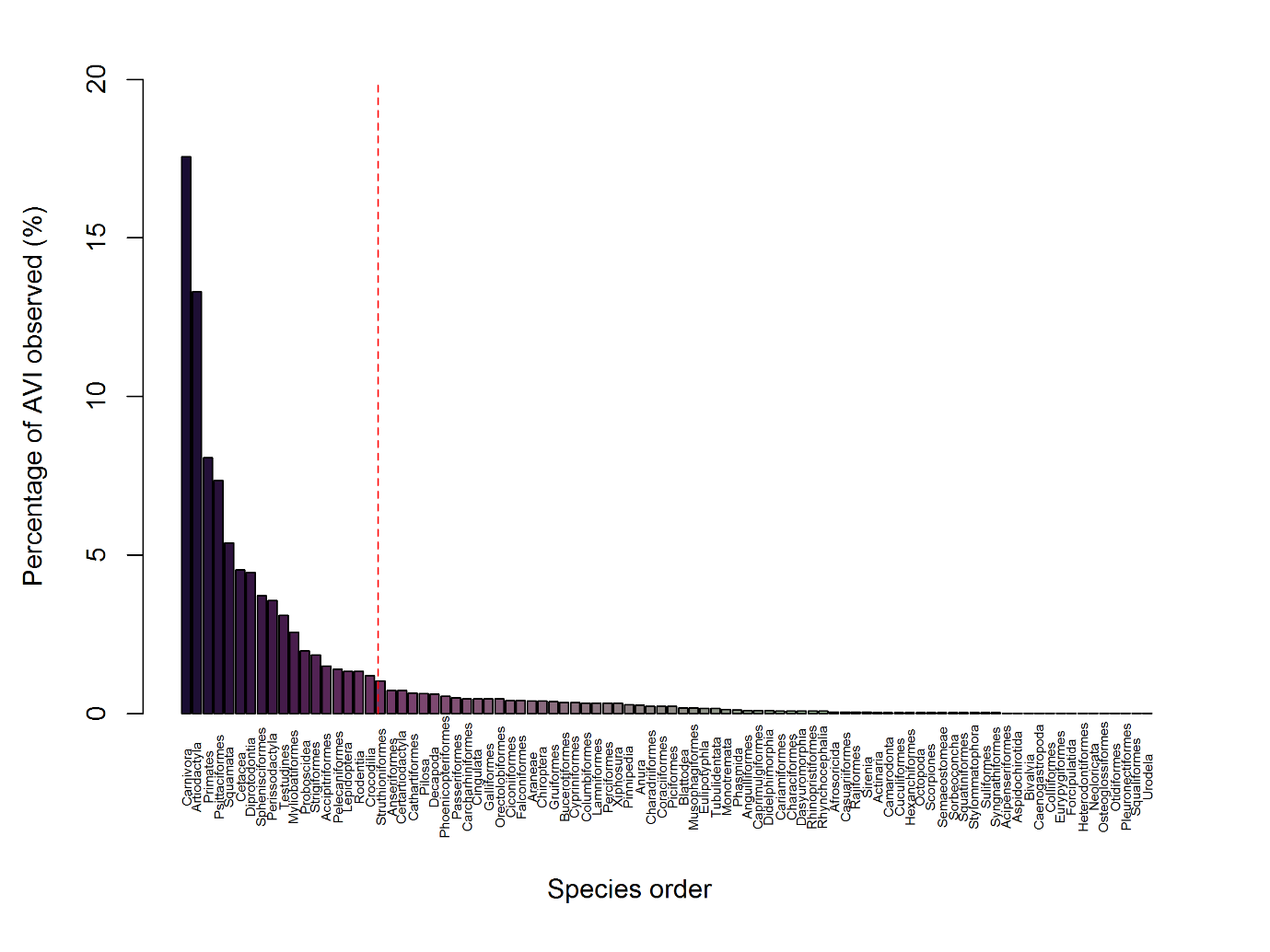
**.

**Figure S1.** Percentage advertised AVIs split by species order. Orders left of the red dashed line were higher in frequency than expected given an even distribution.
